# Performance of T1 mapping vs. T2 mapping for assessing myocardial edema

**DOI:** 10.1186/1532-429X-16-S1-O16

**Published:** 2014-01-16

**Authors:** Janelle Yu, Dominik P Guensch, Kady Fischer, Gobinath Nadeshalingam, Matthias G Friedrich

**Affiliations:** 1 Philippa & Marvin Carsley CMR Centre, Montreal Heart Institute, Montreal, Quebec, Canada; 2 Anesthesiology and Pain Medicine, University Hospital Bern, Bern, Switzerland

## Background

Edema is an early feature of acute myocardial injury. Signal-intensity based analysis of T2-weighted images is established but known to have a high variability. T2 mapping and, more recently, T1 mapping have been proposed for assessing myocardial edema. We investigated the ability to accurately detect myocardial edema following defibrillation with T2 maps and two different T1 mapping sequences, using histology as a standard of reference.

## Methods

Myocardial damage was induced in ten anesthetized pigs by cumulative electric shocks with a total energy of 1000J. Images were acquired five hours after damage with two different T1 techniques (MOLLI, modified look-locker inversion recovery and SASHA, SAturation-recovery single SHot Acquisition) and T2 mapping. In addition, six pigs underwent the same protocol without injury. After euthanasia, tissue was randomly sampled from segments with injury by at least one of the mapping approaches. At least two random biopsies were also obtained from the control animals. In a blinded fashion, hematoxylin and eosin stains of these samples were assessed for intercellular and interstitial edema using a semi-automatic planimetry technique. Cellular edema was quantified using a mean area of 5-8 muscle fibers in short axis view. To analyze for interstitial edema, a semi-quantitative planimetric analysis of three representative areas of each sample in 20× magnification was performed. ROC curves were generated and compared to histology as the gold standard.

## Results

There was consistency between MOLLI and SASHA maps vs. histological results of the mean cell area and percentage interstitial space. The comparison of MOLLI to mean cell area was found to have a high negative predictive value with a sensitivity of 0.90 and specificity of 0.71 (p < 0.01). As compared to percentage interstitial space, MOLLI had a sensitivity of 0.95 and specificity of 0.63 (p < 0.01). For mean cell area and percent interstitial space the sensitivity of T2 mapping was 0.91 yet specificity was low (0.35, p = 0.19).

## Conclusions

Compared with histology, T1 mapping sequences appear to be more accurate in correctly identifying myocardial edema than T2 mapping.

## Funding

Funding is provided by the Montreal Heart Institute Foundation and the Canadian Foundation for Innovation.

**Figure 1:**
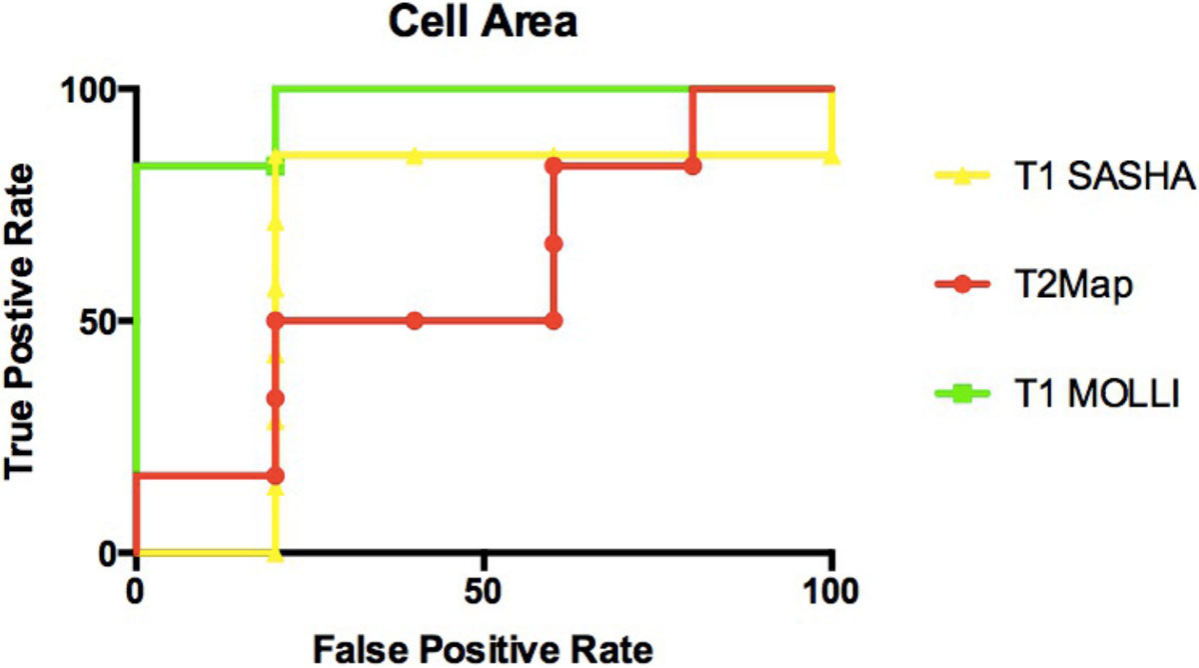
**ROC curves of T2 mapping, T1 MOLLI and T1 SASHA using mean cell area in a short axis view as a gold standard for cellular edema**.

**Figure 2:**
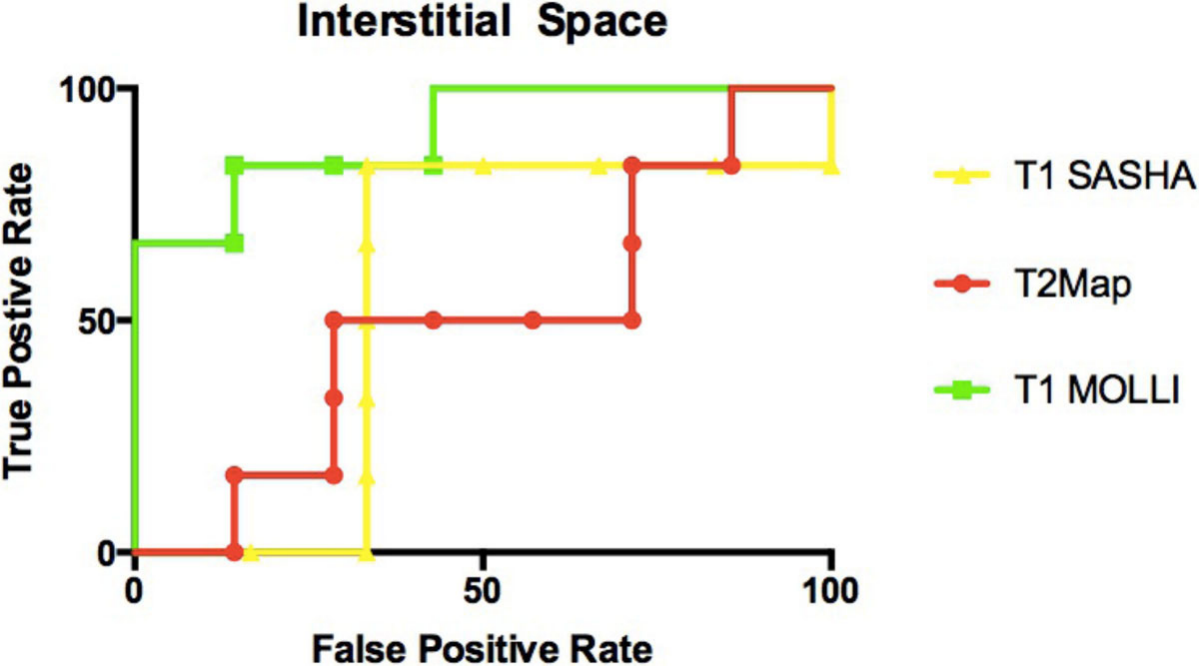
**ROC curves of T2 mapping, T1 MOLLI and T1 SASHA using percent interstitial space as a gold standard for interstitial edema**.

